# Expression of S100A8 correlates with inflammatory lung disease in congenic mice deficient of the cystic fibrosis transmembrane conductance regulator

**DOI:** 10.1186/1465-9921-7-51

**Published:** 2006-03-29

**Authors:** Sam Tirkos, Susan Newbigging, Van Nguyen, Mary Keet, Cameron Ackerley, Geraldine Kent, Richard F Rozmahel

**Affiliations:** 1 Department of Pharmacology, University of Toronto, Toronto, Ontario, Canada; 2 Department of Pathobiology, University of Guelph and Ontario Veterinary College, Guelph, Ontario, Canada; 3 University of Western Ontario, London, Ontario, Canada; 4 Lawson Health Research Institute, London, Ontario, Canada; 5 The Hospital for Sick Children, Toronto, ON, Canada

## Abstract

**Background:**

Lung disease in cystic fibrosis (CF) patients is dominated by chronic inflammation with an early and inappropriate influx of neutrophils causing airway destruction. Congenic C57BL/6 CF mice develop lung inflammatory disease similar to that of patients. In contrast, lungs of congenic BALB/c CF mice remain unaffected. The basis of the neutrophil influx to the airways of CF patients and C57BL/6 mice, and its precipitating factor(s) (spontaneous or infection induced) remains unclear.

**Methods:**

The lungs of 20-day old congenic C57BL/6 (before any overt signs of inflammation) and BALB/c CF mouse lines maintained in sterile environments were investigated for distinctions in the neutrophil chemokines S100A8 and S100A9 by quantitative RT-PCR and RNA in situ hybridization, that were then correlated to neutrophil numbers.

**Results:**

The lungs of C57BL/6 CF mice had spontaneous and significant elevation of both neutrophil chemokines S100A8 and S100A9 and a corresponding increase in neutrophils, in the absence of detectable pathogens. In contrast, BALB/c CF mouse lungs maintained under identical conditions, had similar elevations of S100A9 expression and resident neutrophil numbers, but diverged in having normal levels of S100A8.

**Conclusion:**

The results indicate early and spontaneous lung inflammation in CF mice, whose progression corresponds to increased expression of both S100A8 and S100A9, but not S100A9 alone. Moreover, since both C57BL/6 and BALB/c CF lungs were maintained under identical conditions and had similar elevations in S100A9 and neutrophils, the higher S100A8 expression in the former (or suppression in latter) is a result of secondary genetic influences rather than environment or differential infection.

## Background

Cystic fibrosis (CF) is an autosomal recessive disease caused by mutations in the Cystic Fibrosis Transmembrane conductance Regulator (CFTR) gene [1,2]. Clinical manifestations of CF include exocrine pancreatic insufficiency, intestinal obstruction, male infertility and particularly lung disease [3]. To date, over 1000 CF-causative mutations have been identified in CFTR [4].

Lung disease is the leading cause of morbidity and mortality among CF patients, and is increasingly regarded as multifactorial, being a combination of abnormalities in inflammatory response and pathogen clearance, in addition to electrolyte transport and airway surface layer composition [3,5-16]. Due to yet unknown CFTR-dependent processes, CF lung disease presents as a vicious cycle of inflammation and infection, ultimately leading to the destruction of the airways (reviewed in [3,7,17]). A hallmark of the CF lung disease is a massive and inappropriate influx of neutrophils that release profuse amounts of proteases and activated oxygen radicals, resulting in severe pulmonary damage (reviewed in [3,7,17]). Along with the inappropriate influx of neutrophil into the CF airways, a dysregulation in the levels of inflammatory cytokines, including IL-1β, IL-6, IL-8 and TNF-α are detected [10-16], [18-20]. Given that numerous studies have demonstrated heightened or prolonged inflammatory responses [5] and upregulation of inflammatory mediators in presymptomatic or uninfected CF infants [6,8,9,21,22], it remains unclear whether the inflammation precedes infection or is a result of its destructive properties.

Mouse models of cystic fibrosis, containing disruptions of the CFTR gene, show epithelial bioelectric lesions similar to that observed in CF patients [23,24](reviewed in [25]). CF mice also manifest different abnormalities of lung physiology and certain strains, including those congenic for C57BL/6, have been shown to be hypersusceptible to infections with CF-associated pathogens and development of inflammatory disease [26-36], also reviewed in [37]. In addition, lungs of CF mice have been shown to demonstrate altered expression profiles of numerous inflammatory markers [31,38-41], reminiscent of the disease in CF patients. Thus, CF mouse models could thus provide important insight into the pathogenesis and/or pathophysiology of the lung disease in patients.

Previous studies by us and others have described a congenic C57BL/6J CF mouse model (B6-CF) that manifests an inflammatory lung phenotype [26,27,42] to some extent similar to that seen in CF patients. The major pulmonary disease phenotype of these mice presents at roughly 6 months-of-age with inflammation, interstitial fibrosis, loss of non-ciliated cells, bronchiolar mucus retention, alveolar wall thickening and alveolar hyperinflation. At roughly 4 to 5 weeks-of-age B6-CF lungs present a marked influx of neutrophils, which heralds the more advanced inflammatory lesions. This overt lung disease phenotype appears spontaneous in that no precipitating airway pathogen infections are detected either preceding or concurrent to the onset of inflammation. In contrast to the B6-CF animals, congenic BALB/c CF mice (Bc-CF) do not develop any obvious lung disease phenotype, even at later ages [26,27,42].

To gain further insight into the early pathogenesis of the lung disease in B6-CF mice we previously undertook a study to identify genes having differential expression between 20 day-old lungs (before any indications of an abnormal lung phenotype) of B6-CF and age- and sex-matched wild-type sibs maintained in a specific pathogen free environment and free of any detectable lung infection, using Affymetrix GeneChip™ analysis [43]. These studies identified the neutrophil chemokine S100A8 (also known as mMRP8, Calgranulin B or CP-10) (reviewed in [44]) as having roughly 3-fold elevated expression in the B6-CF compared to wild-type lungs [43]. S100A8, along with the related S100A9 (also known as MRP14), are members of the S100 calcium-binding protein family involved in regulation of calcium dependent intracellular processes (reviewed in [45]) and act as potent chemokines for neutrophil recruitment to sites of inflammation (reviewed in[44,46,47]). In inflammatory states, expression of S100A8 is co-upregulated with S100A9 [46,48] and reviewed in [44,47,49-51]. Here we report that S100A9 expression shows spontaneous (without detectable infection) and early (before 20 days of age) increased expression in lung neutrophils of both B6-CF and Bc-CF mice, in agreement with an approximate 3-fold increase in the number of resident neutrophils. However, the expression of S100A8 was not elevated in the lungs of Bc-CF mice, whereas those of B6-CF showed elevated expression that appeared to correlate with increased neutrophil numbers. Importantly, no increased levels of either S100A8 or S100A9 were detected in other CF-affected tissue (ileum and liver) of these animals. These results suggest: 1) an early and spontaneous (without any detectable precipitating infection) inflammatory phenotype in the lungs of CF mice, 2) progression to overt lung disease in CF mice corresponds to elevated levels of both S100A8 and S100A9 (or only S100A8), but not S100A9 alone, and 3) a prominent influence of secondary genetic factors on differential regulation of S100A8 expression.

## Methods

### Mouse studies

The B6-CF and Bc-CF mice used for this study and their phenotypes have been described in detail elsewhere [26,27,52,53]. All studies were carried out on 20-day-old mice before any evidence of lung inflammation in the B6-CF animals as previously described [26,27], and personal communication (Dr. G. Kent). To alleviate the severe intestinal lesions resulting in the early death of the congenic B6-CF mice, they were placed on a liquid Peptamen diet from age 18-days until sacrifice, as previously described [54].

Genomic DNA was prepared from tail clips using a salting-out extraction procedure [55]. Briefly, about 2 cm of tail was removed and digested overnight at 55°C with proteinase K (0.5 mg/ml). Proteins were then precipitated with a saturated NaCl solution followed by centrifugation at 13,000 rpm for 10 min. DNA was ethanol precipitated and redissolved in Tris-EDTA buffer. PCR reactions were performed as previously described [54]. Briefly, the wild-type and mutant CFTR alleles were detected in the mice by PCR, using primers specific for the endogenous CFTR locus and for the mutant CFTR locus: Primer A (wild type) 5'-CTGTAGTTGGCAAGCTTTGAC-3'; Primer A (mutant) 5'-ACACTGCTCGAGGGCTAGCCTCTTC-3'; Primer B (wild type and mutant) 5'-CAGTGAAGCTGAGACTGTGAGCTT-3'. The PCR was performed using standardized conditions: 2 mM MgCl^2^, 200 mM dNTPs, 100 nM each primer, 100 ng genomic DNA, and 1 U Taq polymerase. Thermal cycling was carried out for 35 cycles (1 min, 94°C; 1 min, 50°C; 1 min, 72°C). After electrophoresis the PCR products were visualized on an ethidium bromide stained 1% agarose gel.

All mice (CF and wild-type controls) were maintained under stringent Specific Pathogen Free (SPF) conditions in microisolator cages at the Hospital for Sick Children Animal Facility, as previously described [26]. Detailed serological surveillance was continuously performed on the entire colony of CF mice using sentinel animals. Sentinels were placed in open cages adjacent to, and/or in the same cage as, the CF heterozygous breeders for 3 months and then exsanguinated. The sera from these animals was frozen and shipped to the University of Missouri Research Animal Diagnostics Laboratory (Columbia, MO) to be screened for rodent viral pathogens (mouse hepatitis, Sendai, mouse pneumonia, respiratory enteric orphan, ectromelia, Theiler's murine encephalitis, mouse adenoviruses 1 and 2, lymphocytic choriomeningitis, infant mouse enzootic diarrhea, polyoma, and parvovirus), *Carbacillus *and *Mycoplasma pulmonis*. A second group of sentinels (congenic C57BL/6J CF and C57BL/6J heterozygous CF breeders) housed in open cages adjacent to the heterozygous CF breeders were maintained under the same conditions for an additional 6 weeks. Half of these animals were screened as above, while the remaining mice were sent to the Ontario Veterinary College Department of Pathology, University of Guelph (Guelph, Ontario, Canada) for detailed histopathological screening for signs of infections of their lungs, kidneys, heart, spleen, pancreas, salivary glands, jejunum, ileum, colon, brain, seminal vesicles, thymus, and lymph nodes. Lung and jejunal tissue were also routinely cultured for bacteria, and found to be negative for conventional CF lung pathogens (E. *coli*, P. *aeruginosa*, B. *cepacia*, S. *aureus*, as well as Proteus and Streptococcus sp). In addition, histopathological screens were also performed to detect pathogenic infections of the specific lung samples used for RNA preparation. The studies performed did not identify any obvious signs of lung infection in the CF animals; nevertheless it is not possible to completely rule out the presence of any undetected pathogens.

### mRNA quantification

Total cellular RNA was extracted from snap-frozen whole right lung lobes dissected from 20-day old CF and wild-type sibs from both the C57BL/6 and BALB/c strains (8 of each genotype/strain) using the Qiagen RNAeasy™ Midi kit according to the manufacturer's protocol. Sample concentration and purity were determined by measuring optical density at 260 nm and the ratio of 260 nm to 280 nm, respectively. A ratio of absorbance (A_260_/A_280_) between 1.6 and 1.9 was considered acceptable for purity. RNA integrity was assessed by visualization on an ethidium bromide stained 1% agarose gel. One microgram of total cellular RNA from each sample was then treated with 1 unit of amplification grade DNase I (Invitrogen) according to the manufacturer's protocol.

To determine S100A8 and S100A9 mRNA expression levels, 1 μg of DNase I-treated total cellular RNA from the mouse whole lung was reverse transcribed using the Invitrogen Superscript™ II RNase H^- ^Reverse Transcriptase First-Strand Synthesis kit using conditions recommended by the manufacturer. Briefly, 1 μg of DNase I-treated total RNA and oligo(dT)_12–18 _primer were incubated at 65°C for 5 minutes, added to 5X RT buffer, 0.1 M DTT, 10 mM deoxyribose nucleotide triphosphate (dNTP) mix and incubated at 42°C for 2 minutes. Fifty units of Superscript™ II reverse transcriptase was added and the mixture was incubated at 42°C for 50 minutes, 70°C for 15 minutes, then treated with 2 units of RNase H at 37°C for 20 minutes and stored at -20°C. Oligonucleotide primers to amplify the target S100A8, S100A9 and the β-actin cDNA sequences were designed from published cDNA sequences (Genbank ascension numbers S57123, M83219 and X03672, respectively). The primers were chosen to span at least 1 intron to distinguish products resulting from the amplification of cDNA and potentially contaminating genomic DNA. Primer sequences were as follows: S100A8 sense 5'-CCCGTCTTCAAGACATCGTTTG-3' (position 1–22 in the cDNA), S100A8 antisense 5'-ATATCCAGGGACCCAGCCCTAG-3' (position 347–326 in the cDNA), S100A9 sense 5'-CCCTGACACCCTGAGCAAGAAG-3' (position 120–141 in the cDNA), S100A9 antisense 5'-TTTCCCAGAACAAAGGCCATTGAG-3' (position 453–430 in the cDNA), β-actin sense 5'-GTGGGCCGCCCTAGGCACCAG-3' (position 183–203 in the cDNA), β-actin antisense 5'-CTCTTTGATGTCACGCACGATTTC-3' (position 722–699). The expected size of the PCR products was 347 bp for S100A8, 333 bp for S100A9 and 539 bp for β-actin. Multiplex PCR amplification was performed using 1/20 of the total cDNA reverse transcribed from each sample. A total reaction volume of 20 μL also contained 200 μM dNTP mix, 150 μM MgCl_2_, 10 mM Tris-HCl (pH 8.3), 50 mM KCl, 2.5 units of *Thermus aquaticus *(Taq) DNA polymerase (Fermentas) and 2.5 ng/μL of both sense and antisense oligonucleotide primers for the target (either S100A8 or S100A9) and the endogenous standard (β-actin). Four reactions were run in parallel for each sample in a Perkin Elmer Applied Biosystems Geneamp Thermocycler 9700, using a hot-start protocol where *Taq *polymerase was added to reaction mixtures after an initial denaturation step at 94°C for 3 minutes. The reactions were cycled at 94°C for 30 seconds (denaturation), 65°C for 30 seconds (annealing) and 72°C for 30 seconds (extension). Equal volumes of the PCR reaction were removed at cycles 19, 21, 23 and 25. Fifteen microliters from each PCR reaction were loaded unto an ethidium bromide stained 1% agarose gel and documented with a Kodak EDAS 290 electrophoresis documentation system. Band intensities (and thus starting product levels) of the target relative to control were measured using the program NIH Image^® ^. Band intensities of PCR products (S100A8, S100A9 and β-actin) were plotted against cycle number in order to determine the exponential phase of amplification. For each sample, the S100A8 and S100A9 multiplex RT-PCR product band intensities after 21 cycles of amplification were normalized to that of the β-actin produced in the same tube and the mean of the four runs was calculated to obtain relative expression levels. All measurements for expression were performed with the investigator blinded to mouse strain and genotype.

### Neutrophil counts

To ascertain relative neutrophils numbers in the lungs of the different mice (B6-CF, Bc-CF, and their wild-type sibs) the left lung lobes of 7 animals of each strain and genotype were harvested, inflated, and infused with 10% buffered formalin. After overnight fixation in formalin the lobes were cut into 4 separate sections (from top to bottom of the lobe to maximize representation of the specimen), embedded in paraffin blocks and sectioned to a thickness of 4 μm followed by Hematoxylin & Eosin (H&E) staining for visual inspection and counts of neutrophils (recognized by their characteristic multi-lobed nuclei) by an experienced pathologist blinded to strain, genotype and expression status. For each of the 4 lung sections from each animal, the number of neutrophils in 6 distinct and randomly chosen fields was counted and the average of the 6 was calculated for that lung section. Thus, a total of 24 distinct sections of each lung from 7 mice (168 total independent fields) of each genotype and strain were counted to arrive at a representative measure of neutrophil content for each group of animals.

### RNA in situ hybridization

Left lung lobes (4 of each genotype/strain) were inflated, fixed in paraformaldehyde, OCT-embedded and thin-sliced (5 independent sections for each lung) onto aminoalkylsilane-coated slides (SIGMA) followed by air-drying for 2 hrs. Samples were fixed in 4% paraformaldehyde in PBS for 20 min, protein hydrolyzed in 20 μg/ml proteinase K for 7.5 min, and then post-fixed for 5 min in 4% paraformaldehyde in PBS. Tissues were incubated for 10 min in a 0.1 M triethanolamine, 0.5 % acetic anhydride solution. To dehydrate samples, slides were dipped successively in a graded series of ethanol baths before hybridization. Samples were hybridized overnight at 55°C in 50% formamide, 0.3 M NaCl, 20 mM Tris-HCL (pH 7.6), 5 mM EDTA, 10% dextran sulphate, 1.5 × Denhardts, 0.5 mg/ml yeast tRNA, and digoxigenin-UTP-labeled RNA probes. Antisense and sense probes were prepared by in vitro transcription, using T7 RNA polymerase, from a 347 bp sequence (nucleotides 1–347) of S100A8, and a 333 bp sequence (nucleotides 120–453) of S100A9, of HindIII linearized pCR2.1 (Invitrogen) vector with S100A8 and S100A9 inserted in both orientations into the BamHI/HindIII sites of the multiple cloning region. Following hybridization, slides were soaked for 15 min in 0.1 M maleic acid and 0.15 M NaCl, then for 1 hr in a 1% Boehringer blocking reagent solution in 0.1 M maleic acid and 0.15 M NaCl. Bound probes were detected by exposing samples to alkaline phosphatase-conjugated anti-digoxigenin antibodies for 1.5 hrs and slides were then washed in 0.1 M Tris (pH 9.5), 0.1 M NaCl, 50 mM MgCl_2 _for 10 min. The substrate, nitro blue tetrazolium/5-bromo-4-chloro-3-inolyl phosphate (Invitrogen), was added to the samples and the color reaction was allowed to develop overnight. All samples were hybridized to both anti-sense and sense (negative control) probes to ensure specific signal detection. The number of positive-staining neutrophils in 5 independent fields for each section was counted and the average taken as representative of that lung.

### Statistical analysis

All statistical comparisons were performed using non-parametric Mann-Whitney Tests (2-tailed) and Spearman Rank Correlation tests, as appropriate. Data is plotted as the median with interquartile ranges.

## Results

### Lung-specific upregulation of S100A8 and S100A9 in CF mice

**Figure 1:**
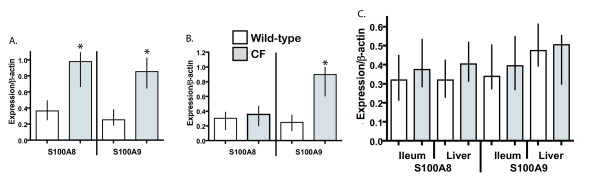
Semi-quantitative reverse-transcriptase PCR of S100A8 and S100A9 expression relative to β-actin in the lungs of **A. **congenic C57BL/6 CF and wild-type mice, **B. **congenic BALB/c CF and wild-type mice, (n = 8 for each strain/genotype), and **C. **ileum and liver of CF and wild-type mice from both strains (n = 5 for each strain/genotype). White and gray bars represent wild-type and CF samples, respectively. Median with 25% and 75% intervals are shown. An asterisk (*) denotes a significant difference between the wild-type and CF samples (p ≤ 0.05).

We had previously reported a roughly 2.5-fold elevation of S100A8 expression in the lungs of 20 day-old B6-CF mice, as ascertained through an Affymetrix GeneChip experiment [43]. To confirm this increase in expression, semi-quantitative RT-PCR experiments were undertaken. As shown in Fig. 1A, analysis of the expression data showed significantly (p ≤ 0.005, two-tailed Mann-Whitney test) elevated expression of S100A8 (~2.5-fold) in the lungs of B6-CF mice compared to their wild-type sibs, in agreement with the microarray data. Since the expression of S100A8 may be coordinately regulated with its heterodimerization partner S100A9, the expression level of S100A9 was next investigated in these lungs. As shown in Fig. 1A, expression of S100A9 also had roughly 2.5-fold higher expression in the lungs of B6-CF mice compared to their wild-type littermates (p ≤ 0.005), confirming a coordinate increase in levels of the two S100 mRNAs in B6-CF lungs. In contrast, similar studies of 20 day-old Bc-CF lungs, which do not progress to the inflammatory lung disease phenotype, maintained under identical conditions showed no significant increase in S100A8 expression (p ≤ 0.5), although expression of S100A9 was significantly elevated (p ≤ 0.001) in a manner similar to that of the B6-CF samples (Fig. 1B). A significant increase of S100A8 levels was detected in all 8 B6-CF lungs examined, while none of the B6–WT, Bc-CF or Bc-WT lung samples from identical environments showed a marked elevation. Furthermore, no significant difference in either S100A8 or S100A9 expression levels was detected in non-airway tissue, including the ileum (tissue most severely affected in CF mice) or liver of CF compared to wild-type animals of both C57BL/6J and BALB/cJ strains (p ≤ 0.5, five mice for each group), as shown in Fig. 1C, indicating that increased levels of S100A8 and S100A9 expression were lung specific.

These results indicate an early and specific increase of both S100A8 and S100A9 expression levels in lungs of B6-CF mice in contrast to Bc-CF lungs in which only S100A9 expression levels were elevated.

### Elevated neutrophils in CF mouse lungs

**Figure 2:**
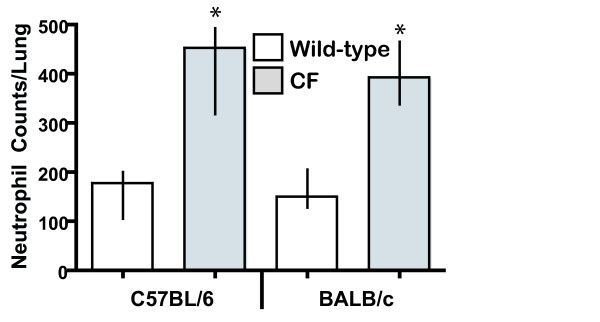
Neutrophil counts. The average number of neutrophils in the lungs of 20 day-old congenic C57BL/6 and BALB/c wild-type (white bars) and CF (gray bars). The average number of neutrophils per lung section (n = 4 levels per left lobe, n = 6 fields for each level) from 7 animals per strain/genotype is represented. Median with 25% and 75% intervals are shown. An asterisk (*) denotes a significant difference between the wild-type and CF samples (p ≤ 0.05).

To assess the basis of the differential S100A8 and S100A9 levels, the number of resident neutrophils (primary sites of S100A8 and S100A9 expression) between the lungs of 20 day-old B6-CF, Bc-CF and their wild-type sibs were next quantified as described in Materials and Methods. As shown in Fig. 2, the B6-CF mice showed a significant 2.6-fold increase in resident neutrophils in their airways and interstitium, compared to their wild-type sibs (p ≤ 0.001). Similarly, Bc-CF mice had a significant roughly 3-fold increase in neutrophil numbers compared to their wild-type sibs (p ≤ 0.005). Thus, since neutrophils are the primary site of expression of S100A8 and S100A9, and the B6-CF and Bc-CF lungs showed an almost 3-fold increase in neutrophil count, respectively, the elevation of S100A9 in both strains of CF lungs, and in the B6-CF lungs for S100A8, likely corresponds to the increased neutrophil numbers. Supplementary assessment of the correspondence between neutrophil numbers and S100A8/S100A9 expression levels per sample was performed by Spearman Rank Correlation analyses, which further supported a likely relationship (p ≤ 0.005 for all results, with the exception of S100A8 in the Bc-CF lungs (p ≤ 0.5)). The lack of correlation between S100A8 levels and neutrophil numbers in the Bc-CF lungs suggests a suppression of its expression in neutrophils in this strain. An assessment of resident macrophage between the B6-CF and B6–WT lungs did not detect a significant difference in numbers (data not shown), suggesting that this early course of the inflammatory lung phenotype appears to be limited to neutrophils.

### Localization of S100A8 and S100A9 lung expression

**Figure 3:**
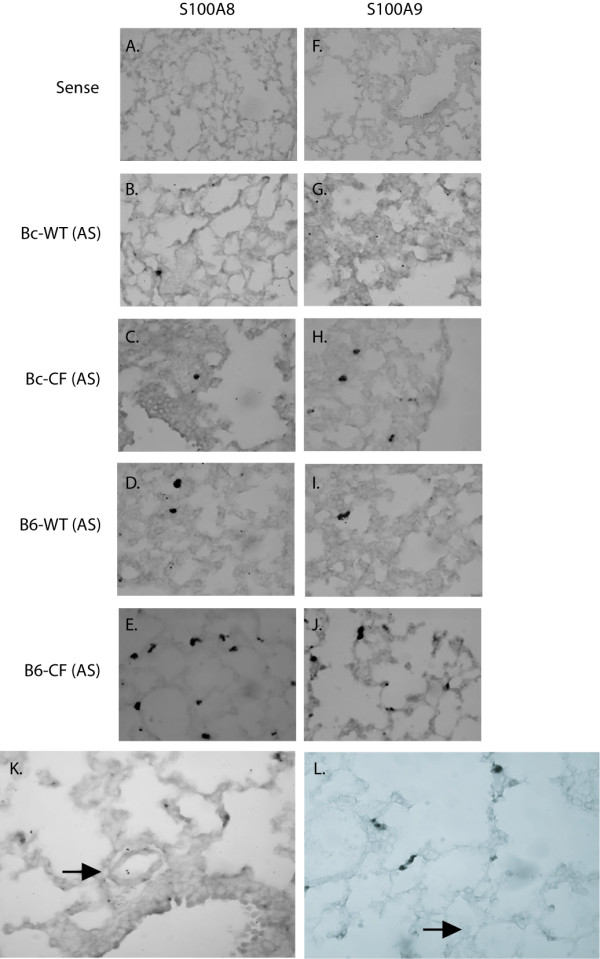
RNA *in-situ *hybridization of lungs with S100A8 and S100A9. Panels **A **and **F **represent 20 day-old congenic C57BL/6J CF lung sections stained with a sense probe for S100A8 and S100A9, respectively. The panels are representative of S100A8 antisense hybridized sections of 20 day-old Bc-WT (**B**), Bc-CF (**C**), B6–WT (**D**) and B6-CF (**E**) mouse lungs, and S100A9 antisense probe hybridized sections of 20 day-old Bc-WT (**G**), Bc-CF (**H**), B6–WT (**I**) and B6-CF (**J**) mouse lungs. Panels **K **and **L **show a absence of staining for S100A8 in endothelial cells and macrophage of 20 day-old B6-CF lungs, respectively. Panels A-J are shown at 40X magnification and panels K and L are at 60× magnification.

**Figure 4:**
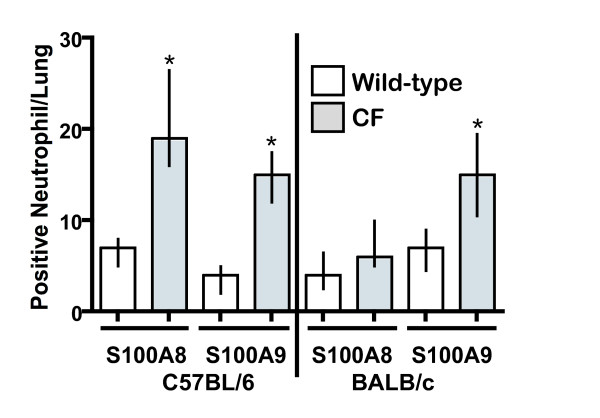
Counts of positively staining neutrophils for S100A8 (A8) and S100A9 (A9) in lungs of 20 day-old congenic C57BL/6 and BALB/c wild-type (white bars) and CF (gray bars) mice. The values represent the average number of positive-staining neutrophils from 4 mice of each strain/genotypes with 5 independent sections and 5 fields from each section for each. Median with 25% and 75% intervals are shown. An asterisk (*) denotes a significant difference between the wild-type and CF samples (p ≤ 0.05).

To confirm the specific cell types conferring S100A8 and S100A9 expression, RNA *in situ *hybridization of lung sections taken from 20-day old Bc-CF, B6-CF mice and their wild-type sibs was performed. As expected, hybridization of the lung sections with S100A8 and S100A9 sense probes showed no positively staining cells (Fig. 3A and 3F, respectively). Both the S100A8 and S100A9 antisense probes detected staining only in a small number of scattered neutrophils in the B6–WT lungs (Fig. 3D and I, respectively), which did not appear significantly different in number to that seen in the Bc-WT lungs (Fig. 3B and 3G, respectively). Hybridization of Bc-CF lung sections with S100A8 (Fig. 3C) only rarely detected positively staining cells, similar to their Bc-WT sibs, whereas S100A9 (Fig. 3H) detected markedly more staining cells, which were identified morphologically as neutrophils. In contrast, both the S100A8 and S100A9 probes detected significantly higher numbers of positive neutrophils in the B6-CF lungs (Fig. 3E and 3J, respectively). Summation of the number of total S100A8 and S100A9 staining neutrophils per B6 lung assessed revealed an almost 3-fold higher number of positive cells in the CF compared to wild-type lungs (p ≤ 0.01, in both cases) (Fig. 4), in agreement with the increased numbers of neutrophils identified by morphometric measures and the increase in levels of expression in the lungs. A similar determination of the number of total S100A8 and S100A9 staining cells in the samples from the Bc strain showed no significant difference (p ≤ 0.20) with S100A8; however, the S100A9 probe detected a significant (p ≤ 0.01) increase positively-staining neutrophils (Fig. 4), in agreement with the morphometric measures and increased whole lung expression.

Importantly, other cell types reported to have inducible expression (vascular endothelial cells and macrophage) were negative for S100A8 staining in B6-CF lungs (Figure 3K and 3L, respectively), indicating that its increased levels in whole lungs were not the effect of induction in such cells, but exclusively due to the increased numbers of expressing neutrophils. However, due to limitations inherent of RNA *in situ *hybridization it was not possible to quantitate S100A8/S100A9 expression levels on a per cell level.

## Discussion

Differential disease states between distinct congenic mouse strains harboring identical mutations and maintained in a common environment provides a powerful means for identifying secondary genetic factors that have influence disease phenotypes. Here we report that the lungs of 20-day old congenic C57BL/6J CF mice, that progress to overt inflammatory disease, maintained in a sterile environment have elevated numbers of neutrophils and a corresponding increased level of both S100A8 and S100A9, which is not detected in other CF-affected tissues (ileum and liver). In contrast, the lungs of 20-day old congenic BALB/cJ CF mice, which do not develop any obvious inflammatory phenotype, housed with the congenic C57BL/6J CF animals, had no increase in S100A8 levels, although resident airway neutrophil numbers and S100A9 levels were similarly elevated.

S100A8 (calgranulin A, MRP8) and S100A9 (calgranulin B, MRP14) are small cytoplasmic proteins (members of the S100 family of the EF hand calcium-binding proteins [56]) that are expressed principally, constitutively and coordinately by circulating neutrophils and monocytes but not normally in tissue macrophages or lymphocytes [57]. The two proteins make up roughly 30% of the cytosolic protein in these cells [58] and support distinct functions (both as monomers and homodimers), as well as forming calprotectin (S100A8/S100A9 heterodimer) in the presence of Ca^2+^, with potentially different function(s). Although an understanding of the complete role(s) of each of S100A8, S100A9 and calprotectin is currently lacking [57,59] diverse functions that could impact on CF lung disease have been attributed to them, including calcium sensing [60], cell differentiation, arachidonic acid metabolism [61,62], as well as leukocyte and monocyte endothelial microvascular adherence, transmigration and retention [63-69]. Moreover, calprotectin is implicated in bacteriostasis (reviewed in [44-46,51,70]), possibly by sequestering Zn^2+ ^[71-78] as well as inhibiting the adhesion of bacteria to mucosal epithelial cells [79]. S100A8's important role in regulating inflammatory processes is clearly indicated in S100A8-null mice, where loss of immunoprotection from invading maternal cells results in embryonic death shortly after implantation [80].

During chronic inflammatory conditions, including that underlying CF lung disease, S100A8 and S100A9 are coordinately upregulated and secreted into the extracellular milieu [57,81], and their products elevated in the serum of patients [82-87], (reviewed in [50]). Likewise, coordinate regulation of S100A8 and S100A9 is also observed in neutrophils where absence of S100A9 leads to a coordinate loss of S100A8 expression [60,88]. However, the concise mechanisms of regulatory controls that underlay S100A8 and S100A9 expression are unclear, although they are known to be complex and involve proinflammatory mediators including lipopolysaccharides [89], TNF, IFN-γ and IL-1 [90,91].

The results of the present study are important to further understand the basis and pathogenesis of the inflammatory lung phenotype of CF mice, its distinction among different congenic strains and possibly having implications to understanding airway disease of CF patients. Several important points can be drawn from these results. First, these results provide further support for the increasingly prevalent notion of spontaneous inflammation of the CF airways. This conclusion is supported by: 1) the early incidence of elevations in S100A8 and S100A9 expression along with resident neutrophil influx, 2) the fact that the mice were maintained in sterile environments without detectable lung pathogens, and 3) elevated S100A8 levels were detected in the B6-CF lungs but not Bc-CF airways maintained in identical environments.

Second, since S100A8 and S100A9 act as potent leukocyte chemokines and their elevation at 20-days of age are the earliest reported signs of a lung inflammatory phenotype in CF mice, this elevation may be directly responsible for eliciting the massive neutrophil influx observed in 4–5 week old B6-CF lungs [26,27,42].

Third, these results implicate S100A8 alone or both S100A8/S100A9 (calprotectin), but not S100A9 alone, as having a possible role in progression of the inflammatory lung phenotype in CF mice.

Finally, since both the B6-CF and Bc-CF mice were maintained in identical environments, the differential levels of S100A8 expression between the two strains is likely influenced by secondary genetic factors acting on neutrophils (either intrinsically or through the pulmonary interstitial milieu) to either suppress or upregulate its expression in the Bc or B6 strain, respectively, rather than the effect of differential environmental exposures or infection status. However, since the elevated levels of S100A8 in the B6-CF lungs agrees with the corresponding increased population of neutrophils and no expression was detected in inducible cells (endothelial and macrophage), it is more likely that its expression is being suppressed in the Bc strain as opposed to B6-CF, which maintains expression in resident neutrophils. Since S100A8 is normally expressed in circulating but not interstitial neutrophils [58], a possible explanation for the differential S100A8 levels is that B6-CF neutrophils do not properly recognize or transition to the resident milieu of the CF lung, or their mechanism of suppression may be compromised; thereby, B6-CF neutrophils fail to properly down-regulate S100A8 expression once they leave circulation and enter the lung interstitium, which may constitute a basic defect of the neutrophils or lung in the absence of CFTR function. In this regard, further studies of differences between the B6-CF and Bc-CF lungs in terms of signaling pathways and the mechanisms underlying the neutrophil phenotype transition from circulatory to interstitial, as well as the effect of differential lung milieus on this transition will be required to ascertain the mechanistic basis of this defect.

The results of this study extend on two previous reports of S100A8 overexpression in the lungs of distinct CF mouse lines [31,38]. In the first study by Thomas et al. [31], a constitutive 4-fold overexpression of S100A8 was detected in the lungs of CF mice homozygous for the G551D mutation (in which a spontaneous lung inflammatory phenotype has not been reported) compared to controls. Although expression of S100A9 was not investigated, the results suggested that CF pathology relates to abnormal regulation of the immune system. Importantly, however, this report documented significant variations in basal expression of S100A8 between individual G551D CF lungs, and since these mice were of a mixed 129/Sv × CD1 strain the differences was attributed to genetic variations. It is thus possible that the same genetic factor(s) conferring marked differences in S100A8 expression between congenic C57BL/6 and BALB/c CF lungs correspond to those of the former study, and that the consistent overexpression inherent to the congenic lines (as opposed to the variability of the mixed background) are necessary for the clear and consistent detection of a lung inflammatory phenotype. In the study by Xu et al. [38], a series of microarray analyses were performed to identify differential gene responses to the loss of CFTR in the lungs of FVB/N X C57BL/6 mixed background mice. Of the multiple genes identified as having significantly up- or down-regulated expression in the CF lungs, both S100A8 and S100A9 were found to be roughly 2-fold elevated. However, the specific cells conferring the overexpression and its possible effect on a lung inflammatory phenotype were not investigated. Moreover, since these studies were similarly performed on mixed background mice that would likely also have marked variability in S100A8 and/or S100A9 levels, the potential effect of the overexpression on the lung phenotype could not be readily assessed, in contrast to the strictly controlled aspects of the present investigation.

The results presented here justify additional studies to clarify the role of S100A8 overexpression on the pathogenesis and/or progression of the CF lung inflammatory disease, and, in particular, the possible effect of S100A8 inhibition.

## Conclusion

Taken together, these results derived from genetically-defined CF mice maintained in strict controlled environments provide further support for an early and spontaneous induction of inflammation in lungs devoid of the cystic fibrosis transmembrane conductance regulator, and suggest that S100A8 may play a prominent role. Moreover, since similar elevations of S100A8/S100A9 are detected in CF patients, these results also provide justification for the application of congenic C57BL/6J CF mice as a potential model to gain insight into the pathogenesis of lung disease of CF patients and potential therapeutic avenues.

## Abbreviations

CF: cystic fibrosis

CFTR: cystic fibrosis transmembrane conductance regulator

B6-CF: congenic C57BL/6 CF mice

Bc-CF: congenic BALB/c CF mice

## Competing interests

The author(s) declare that they have no competing interests.

## Authors' contributions

ST performed the majority of the studies, particularly the lung dissection, quantitative RT-PCR, RNA *in situ *hybridization, neutrophil counts and drafting of the manuscript. SN assisted in morphometric analysis, neutrophil and macrophage counts and lung histopathology. VN assisted in RNA preparation, interpretation of quantitative RT-PCR and RNA *in situ *hybridizations. MK performed mouse colony maintenance and genotyping. CA assisted in lung neutrophil and macrophage analysis, measurements and interpretation. GK provided the mice and pathogen monitoring/status and interpretation. RFR designed and supervised the study, and revised the final manuscript.
